# Time to reflect is a rare and valued opportunity; a pilot of the NIDUS‐professional dementia training intervention for homecare workers during the Covid‐19 pandemic

**DOI:** 10.1111/hsc.13737

**Published:** 2022-02-06

**Authors:** Daniel Kelleher, Kathryn Lord, Larisa Duffy, Penny Rapaport, Julie Barber, Jill Manthorpe, Monica Leverton, Briony Dow, Jessica Budgett, Sara Banks, Sandra Duggan, Claudia Cooper

**Affiliations:** ^1^ Centre for Applied Dementia Studies University of Bradford Bradford UK; ^2^ Division of Psychiatry University College London London UK; ^3^ Statistical Sciences University College London London UK; ^4^ NIHR Policy Research Unit on Health and Social Care Workforce King’s College London London UK; ^5^ National Ageing Research Institute University of Melbourne Melbourne Australia; ^6^ Alzheimer’s Society Research Network London UK; ^7^ Camden and Islington NHS Foundation Trust London UK

**Keywords:** dementia care, homecare, independence, interventions, person‐centred care, training

## Abstract

Most people living with dementia want to continue living in their own home for as long as possible and many rely on support from homecare services to do so. There are concerns that homecare often fails to meet the needs of clients with dementia, but there is limited evidence regarding effective interventions to improve its delivery for this client group. We aimed to assess whether a co‐designed, 6‐session dementia training intervention for homecare workers (NIDUS‐professional) was acceptable and feasible. Facilitated training sessions were delivered over 3 months, followed by 3, monthly implementation meetings to embed changes in practice. Two trained and supervised facilitators without clinical qualifications delivered the intervention via group video‐calls during Oct 2020–March 2021 to a group of seven homecare workers from one agency in England. Participants provided qualitative feedback 3‐ and 6‐months post intervention. Qualitative interview data and facilitator notes were integrated in a thematic analysis. Adherence to the intervention and fidelity of delivery were high, indicating that it was acceptable and feasible to deliver in practice. Thirty of a possible 42 (71.4%) group sessions were attended. In our thematic analysis we report one over‐arching theme: ‘*Having time and space to reflect is a rare opportunity’*. Within this we identified four subthemes (Having time to reflect is a rare opportunity; Reflecting with peers enhances learning; Reflection and perspective taking can improve care; Recognising skills and building confidence) through which we explored how participants valued the intervention to discuss their work and learn new skills. Attendance was lower for the implementation sessions, perhaps reflecting participants’ lack of clarity about their purpose. We used our findings to consider how we can maintain positive impacts of the manualised sessions, so that these are translated into tangible, scalable benefits for people living with dementia and the homecare workforce. A randomised feasibility trial is underway.

**What is known about this topic?**

- People living with dementia often rely on paid homecare workers to support their independence.
- The homecare workforce is undervalued and under‐supported, and few homecare workers receive dementia‐specific training.
- There have been few trials of interventions to support homecare workers and none to date in the UK that have been co‐designed with homecare workers and people with lived dementia experience.

**What this paper adds?**

- Our intervention was acceptable and feasible to deliver in one agency, by facilitators without clinical training, using remote delivery.
- Homecare workers valued the intervention groups as a rare opportunity to have time and space to reflect on their work, reporting increased confidence and finding the interactive, group‐based discussions particularly helpful.

## 1 INTRODUCTION

An estimated 850,000 people live with dementia in the United Kingdom (UK), two‐thirds in their own homes (Prince et al., 2014). Most want to continue living as independently as possible and are often supported to do so by family carers and paid homecare workers (Lord et al., 2020). An estimated 400,000 people with dementia and their families rely on homecare services for support (Carter, 2016). For people living with dementia without a regular family carer, homecare services are often their only support. The Alzheimer's Society's (Carter, 2016) *Fix Dementia Care* campaign found that only 2% of people affected by dementia believed homecare workers had sufficient dementia training and nearly half (49%) of people affected by dementia disagreed that ‘homecare workers understand the specific needs of people with dementia’.

Interventions have improved quality of life and quality of care for care home residents living with dementia (Ballard et al., 2020; Lawrence et al., 2016), but evidence regarding effective interventions to improve homecare for people living with dementia is limited (Cooper et al., 2017). Homecare workers often work alone in clients’ homes, so their role and associated training needs are somewhat different to staff working in teams or building‐based communal settings. Heavy workloads and tight schedules are a common barrier to staff training for this population (Su et al., 2021).

Two randomised controlled studies have evaluated dementia training interventions aimed specifically at homecare workers. A 12‐week dementia care training programme in Taiwan improved dementia care knowledge, attitude, and competence of homecare workers (Su et al., 2021). The programme combined e‐learning, and daily online support from a homecare supervisor, who also led monthly face‐to‐face peer support groups. Research suggests that peer support (face‐to‐face or virtual) could be especially important for homecare workers, who often work alone (Yeh et al., 2019). In a study in Japan, the Behaviour Analytics and Support Enhancement (BASE) programme trained homecare workers in a 2‐day course to explore unmet needs and address ‘challenging behaviours’ of people living with dementia, reporting a significant reduction in ‘challenging behaviour’ 6 months after implementation (Nakanishi et al., 2018).

The NIDUS‐professional (New Interventions for Independence in Dementia Study) training intervention is, to our knowledge the first training and support intervention for homecare workers to be co‐designed by this staff group, their managers, health professionals, people living with dementia and their family carers (Lord et al., 2021) in the UK.

Our objective was to assess how acceptable and feasible a group video‐call training and support intervention was to deliver in one homecare agency and whether the NIDUS‐professional intervention is acceptable and feasible to deliver in practice, from homecare worker, homecare manager, and intervention facilitator perspectives; and how homecare workers perceived its value to them and their work.

## 2 METHOD

### 2.1 Design

We delivered a single pilot of the NIDUS‐professional intervention with qualitative evaluation at 3 months (interviews with homecare workers, homecare manager and intervention facilitators) and 6 months (brief telephone follow‐up interview with homecare workers and facilitator reflective logs) post‐intervention.

### 2.2 Ethical approval and trial registration

London‐Camden and King's Cross National Research Ethics Committee approved the study (20/LO/0567); and we registered the protocol (ISRCTN15757555).

### 2.3 Sampling and participants

We recruited homecare workers from one homecare agency in England. The NIDUS programme manager (LD) initially approached the homecare agency training manager, with whom the NIDUS team had a pre‐existing relationship. The training manager identified and approached potential participants, provided them with a study information sheet, and asked for their permission for a researcher to make contact. We included homecare staff who were providing hands‐on care for at least one client with dementia, who understood spoken English, and gave written informed consent to participate.

### 2.4 The NIDUS‐professional intervention

The NIDUS‐professional intervention is a 6‐session manualised training programme for homecare professionals working with people living with dementia. Initially designed as a face‐to‐face group training programme, NIDUS‐professional was adapted for remote delivery via video‐call in response to the Covid‐19 pandemic. The group sessions for 6–8 homecare workers last 60–75 min and focus on exploring practical changes that they can try out to support their client's independence and developing self‐care strategies to manage job stresses (see Table 1 for topics covered).

**TABLE 1:** NIDUS‐professional session topics and intervention components

	*Session 1 ‐ You and your role: looking after yourself as care workers and ways to do this*
1	Welcome & session overview
2	Valuing your role as a homecare worker
3	What is dementia/How does dementia affect your clients
4	Looking after yourself and managing stress
5	End of session: relaxation exercise & putting it into practice
	*Session 2 ‐ Building positive relationships: getting to know the person with dementia and how important this is in providing care*
6	Welcome & session overview
7	Getting to know your clients
8	Understanding how it feels to live with dementia
9	Communicating with your clients
10	End of session: relaxation exercise & putting it into practice
	*Session 3 ‐ The ‘DICE’ (Describe, Investigate, Create strategies, Evaluate) approach: how to understand your clients’ behaviour, and what they communicate through it*
11	Welcome & session overview
12	The DICE model: 'Describe'
13	Investigate: environment
14	Investigate: feelings
15	Investigate: communication
16	Investigate: physical causes
17	Investigate: dementia symptoms
18	End of session: relaxation exercise & putting it into practice
	*Session 4 ‐ Engaging your clients and trying new strategies*
19	Welcome & session overview
20	Supporting dignity & independence
21	DICE continued: Creating and Evaluating Strategies
22	End of session: relaxation exercise & putting it into practice
	*Session 5 ‐ Planning pleasant activities and being a team*
23	Welcome & session overview
24	Planning enjoyable activities
25	Working with families
26	Working as a team
27	End of session: relaxation exercise & putting it into practice
	*Session 6 ‐ Bringing it all together (developing individual and agency action plans)*
28	Welcome & session overview
29	Recap: what it feels like to live with dementia
30	Recap: The DICE model
31	Recap: Planning activities
32	Recap: Relaxation techniques
33	Planning for the future
34	End of session: putting it into practice

This is followed by a 3‐month implementation period, where the facilitators meet monthly with the group to support them to put their learning into practice, share challenges or successes, access peer support and group problem solving, and discuss the practical application of the training.

The NIDUS‐professional intervention is person‐centred. It aims to optimise wellbeing by treating people living with dementia as unique individuals, meeting their psychological needs and respecting their rights. The intervention focuses on peer learning and sharing of experiences, acknowledging and drawing on the homecare workers’ different experiences, and levels of training. We co‐designed the NIDUS‐professional training and support intervention in workshops, with people living with dementia, family carers, homecare workers and managers and health practitioners, using existing interventions (Kales et al., 2015; Livingston et al., 2019; Low et al., 2015; Polacsek et al., 2020), our ethnographic studies (Leverton, 2020; Leverton et al., 2021) and lived experience of co‐designers.

We learned from the Promoting Independence Through quality dementia Care in the Home (PITCH) intervention, a person‐centred intervention designed for and with homecare workers in Australia (Dow et al., 2019). We also drew on materials from a person‐centred intervention designed for care home workers for people living with dementia (Livingston et al., 2019). We presented the PITCH intervention at the first NIDUS coproduction meeting. The PITCH focus on developing empathy by considering how it might feel to have dementia and need homecare was endorsed by the NIDUS coproduction group as being particularly valuable and was therefore incorporated into the NIDUS‐professional intervention.

The intervention co‐design process (Lord et al., 2021) and its theoretical basis, and how this drew on concepts of person‐centred care are described elsewhere (Lord et al., 2020).

### 2.5 Intervention delivery and facilitator training and supervision

NIDUS‐professional was designed to be delivered by graduates in psychology or relevant social science disciplines who do not have formal clinical training to increase scalability. PR and CC trained two members of the research team (KL and DK), without clinical qualifications, but with experience of working with people living with dementia, to facilitate NIDUS‐professional (for facilitator demographics, see Table 2). Facilitator training and intervention delivery were completed remotely, using Zoom video conferencing. Training focused on clinical skills, NIDUS‐professional content, and practical challenges, including adapting to the remote delivery method. Sessions were role‐played by facilitators as part of this training and clinicians (CC and PR) formally assessed these for adherence to the manual.

**TABLE 2:** Intervention facilitator demographics

	Facilitator 1	Facilitator 2
Approximate age (years)	Mid 30s	Late 20s
Gender	Female	Male
First language	English	English
Ethnicity	White British	White British
Highest level of education	PhD	Degree
Years working in dementia field	>10	8

The intervention was delivered to homecare workers (none of whom was involved in co‐producing the intervention) during Oct–Dec 2020. All sessions were recorded to assess fidelity. Participants were offered monthly sessions during Jan–Mar 2021, to support implementation of learning into practice. If participants were unable to join (intervention or implementation sessions), they were offered a ‘catch‐up’ session with one facilitator, either individually or as a small group with other participants. Intervention groups were scheduled during a ‘break’ between shifts when most homecare workers were not visiting clients. We reimbursed the homecare agency for staff costs to ensure that participants were paid for any time spent attending the NIDUS‐professional training and completing the qualitative interviews. Facilitators received weekly supervision with PR to consider and manage challenges around delivery of the intervention. A clinically trained member of the research team (PR/CC) was available for support between supervisions.

### 2.6 Interviews and measures

The researchers obtained written or verbal audio‐recorded consent from eligible homecare workers and collected baseline demographic data (Table 3). Qualitative interviews were conducted with homecare worker participants after they completed their initial training; this included three individual interviews and one ‘group’ interview with two homecare workers (originally intended as a focus group). An additional qualitative interview was also undertaken with the homecare manager. Interviews were conducted by researchers who had not delivered the intervention or collected outcomes with interviewees, using topic guides (Appendix S1), who asked attendees whether and how the intervention had impacted upon client, family carer and homecare workers’ wellbeing and homecare workers’ practice. The topic guide also helped the researchers gather practical and aesthetic feedback regarding the training materials, to aid development in preparation for a larger randomised feasibility trial.

**TABLE 3:** Baseline characteristics of participants

Results are *n* (%) unless specified otherwise	Completed baseline (*n* = 8)	Completed intervention (*n* = 5)
Age (years), median (IQR)	37.5 (34, 41.75)	38 (37, 41)
Gender		
Male	1 (12.5)	1 (20)
Female	7 (87.5)	4 (80)
First language		
English	7 (87.5)	4 (80)
Punjabi	1 (12.5)	1 (20)
Ethnicity		
White British	7 (87.5)	4 (80)
Asian or Asian British: Pakistani	1 (12.5)	1 (20)
Highest level of education		
O levels/GCSEs	3 (37.5)	2 (40)
Vocational (NVQ, GNVQ, BTEC)	3 (37.5)	1 (20)
Degree	2 (25)	2 (40)
Received any training in dementia?		
No	2 (25)	2 (40)
Yes: e‐learning only	4 (50)	1 (20)
Yes: e‐learning & other (dementia awareness)	2 (25)	2 (40)
Job title		
Homecare worker	6 (75)	4 (80)
Homecare worker (supervisor)	2 (25)	1 (20)
Working hours		
Full time	5 (62.5)	3 (60)
Part time	3 (37.5)	2 (40)
Duration working at the agency		
Less than 6 months	4 (50)	3 (60)
1–3 years	3 (37.5)	2 (40)
5–10 years	1 (12.5)	0
Duration working in homecare overall		
Less than 6 months	4 (50)	3 (60)
1–3 years	3 (37.5)	2 (40)
10 years or more	1 (12.5)	0 (0)

DK conducted brief follow‐up telephone interviews with homecare workers at the end of the implementation period (6 months), from which he recorded detailed notes including verbatim participant quotes. The NIDUS‐professional facilitators (KL, DK) participated in a qualitative interview at 3 months and also recorded reflections about their experiences delivering the sessions and implementation period.

### 2.7 Analysis

We described participants’ sociodemographic characteristics and reported adherence (intervention sessions attended, whether in a planned group, catch‐up group, or an individual catch‐up session).

We took a reflective thematic approach to the analysis of qualitative data (Braun & Clarke, 2019). DK and KL systematically and independently coded transcripts of the interviews held after the 3‐month initial training period, and reflective notes from 6‐month follow up telephone interviews. Since DK and KL were intervention facilitators, CC also coded the facilitator interview transcript. The researchers read the transcripts and notes to check for accuracy, anonymity and to familiarise themselves with the data, and then labelled meaningful fragments of text with initial codes. They inductively open and double‐coded material to generate a coding framework. We refined and defined themes through discussion within the NIDUS team.

We integrated findings from the different data sources by exploring how codes from one dataset followed into the other, and vice versa (Moran‐Ellis et al., 2006), developing one interwoven framework. Although we considered data sources equally valid, the majority of material reported comes from the 3‐month interviews as this data form the bulk of the feedback regarding the intervention sessions. The 6‐month telephone interview data were incorporated primarily to provide insights regarding the implementation period. We adhered to COREQ guidelines to ensure methodological rigour of the qualitative analysis (Tong et al., 2007).

To analyse fidelity of NIDUS‐professional delivery, two researchers independently applied checklists to each of the 6 group recordings. We calculated the proportion of expected intervention components (Table 1) delivered. We adopted established thresholds to rate fidelity (Noell et al., 2002): 81%–100% constituted high fidelity, 51%–80% moderate and <50% low fidelity. The researchers rated on a 5‐point scale (1—not at all to 5—very much) whether the facilitator kept the group focused on the manual, and participants engaged, for each intervention component, and for each session, whether the facilitators kept to time.

## 3 RESULTS

### 3.1 Recruitment and retention

**FIGURE 1:**
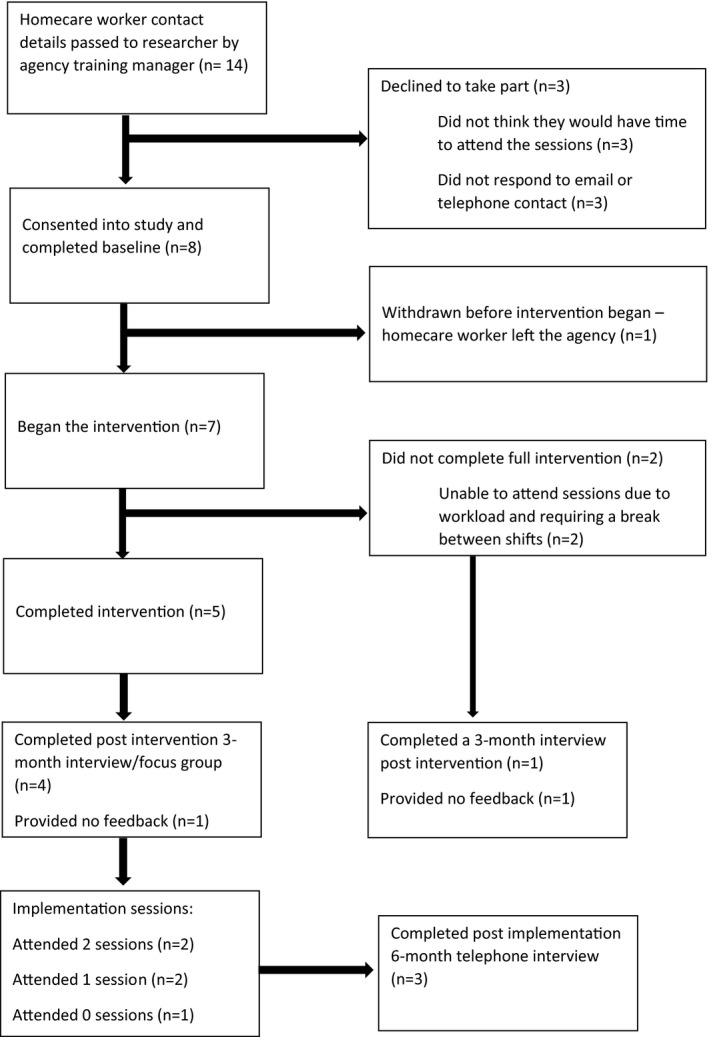
Flow chart of the NIDUS‐Professional Pilot

The homecare agency identified 14 potential homecare worker participants for the researchers to approach. Of these, eight agreed to participate and completed baseline assessments (Figure 1; Flow diagram). Three participants declined to participate, all stating that they were ‘too busy’ to attend the training sessions. The remaining three participants did not respond to contact from the researcher and after several attempts, were assumed to have declined.

Of the eight participants who consented, one decided to leave the agency pre‐intervention, so was no longer eligible. Seven participants therefore began the intervention in October 2020. Of these, five completed all six sessions (via the main group or catch‐up sessions; see Table 4 for attendance details). Two participants attended a single group session and one individual catch up session. Of these two, one withdrew after the second session, stating that they did not have the time to fit the training into their day, between their double shift. The other participant did not wish to withdraw but was unable to attend any further sessions due to workload pressures. Thus overall, 30/42 (71.4%) possible group sessions were attended, and including individual catch‐up sessions 34/42 (80.9%) sessions were attended.

**TABLE 4:** Attendance (group, group catch up, individual catch up, not attended)

Participant (Gender, duration in homecare)		Intervention session						3‐month data	Implementation session			6‐month data
		1	2	3	4	5	6		1	2	3	
P1	Female, less than 6 months	G	G	G	G	GCU	G	Interview	G	x	x	No
P2	Female, 1–3 years	ICU	G	x	x	x	x	No	x	x	x	No
P3	Male, less than 6 months	G	G	G	G	GCU	G	Interview	G	x	x	Interview
P4	Female, 1–3 years	G	G	ICU	G	GCU	G	Group interview	GCU	x	ICU	Interview
P5	Female, 10 years or more	ICU	G	x	x	x	x	Interview	x	x	x	No
P6	Female, 1–3 years	G	G	G	GCU	ICU	G	No	x	x	x	No
P7	Female, less than 6 months	G	G	G	GCU	GCU	G	Group interview	GCU	ICU	x	Interview

Abbreviations: G, attended group; GCU, attended catch up group; ICU, attended individual catch up; x, did not attend.

Of the five participants who completed the intervention, four attended at least one implementation session, as either a group session or an individual catch‐up. Two participants attended one session, and two participants attended two sessions. The fifth participant did not attend any of the implementation sessions due to a change in job role within the agency, meaning she was no longer available to attend. Time pressures and lack of availability were the main reasons that participants reported being unable to attend all three of the implementation sessions. Some participants also stated that since they were so busy, they would only join sessions if they had something that was necessary to discuss with the group.

Sociodemographic characteristics are summarised in Table 3. For most participants, this was their first dementia training course, beyond a mandatory e‐learning module provided by the homecare agency.

### 3.2 Intervention fidelity

Both raters who reviewed audio recordings of the intervention sessions, reported that all 33 intervention session components recorded were delivered; one component, the introduction to session three was not recorded and so could not be evaluated. Thus, overall fidelity (33/34:97%) was in the range specified a priori to be high. Mean fidelity scores across intervention components were assessed as: 4.7 (range 3–5) for ‘Keeping the group focused on the manual/ task’; 4.6 (range 3–5) for ‘Keeping participants engaged’; and 3.75 (range 2–4) for ‘Keeping the session to time’.

### 3.3 Thematic analysis

We identified one overarching theme that responded to our research questions: *‘Having time and space to reflect is a rare and valued opportunity’*. The context of this work, a remote group via video‐call in a pandemic, is evident in narratives. Most participants felt that video conferencing worked well under the circumstances but brought challenges. The remote delivery made it easier to fit the training into their working day, but participants described difficulties in reading social cues and one participant described the impact of this on group dynamics:

We're all waiting and then we all talk. (P7, group interview).

Within our overarching theme, we describe four linked subthemes below.

#### 3.3.1 Subtheme 1: Having time to reflect is a rare opportunity

In the first, *Having time to reflect is a rare opportunity*, we consider how homecare workers perceived having time to step back from the relentless pace of homecare work as rare and valued. The training opportunity was welcomed but increased the intensity of the homecare worker's day. For example, homecare workers attended the sessions from their own homes, during their lunch break, often between shifts. The agency requested that the sessions were held in the early afternoon when few visits were scheduled, but nonetheless participants sometimes missed sessions due to visits over‐running, and when the training over‐ran, missed the end of the session:

I wanted to stay right until the end, because obviously you’d have to say, “I’ve got to get to work now,” and I thought, “I’ve just got to go now and I’ll just hang up.” It’s nice to stay right until the end. (P1, 3‐month, individual interview)

Participants described how client visits are often back‐to‐back, leaving very little time for learning, reflection, and preparation between visits:

Everything is so fast‐paced, like you’ve got one client and then the next client and then the next client… and obviously, because I’m quite new as well, I’m trying to fit everything in and learn. (P7, 3‐month, group interview)

Several participants, including one of the supervisors, suggested that time should be allocated in schedules for the training rather than allowing it to encroach on their break time:

I think maybe if you do it again, we’d plan them to have the evening off, or to cover the tea calls so they were back out later, so they are getting a bit of a break and they’re not feeling like they’ve to fit it in. (P5, 3‐month, individual interview)

Nonetheless, interviewees all spoke positively about the experience:

It didn’t feel like a chore, you felt, I always looked forward to it… it was like, I’m really excited to see what everyone’s been up to this week and see what examples, what we’re going to talk about this week. (P7, 3‐month, group interview)

While most participants spoke more generally about the value of time to reflect on their work, one participant specifically linked this to the opportunity to reflect about dementia care. They suggested that while day to day practicalities of hands‐on care were covered in training, dementia was not a big enough part of their standard training, and suggested that NIDUS‐professional addressed this gap in training provision:

I think you do have really good one‐on‐one training when it comes to, like, how to use a hoist and how to change a catheter and all that, but when it comes to dementia, it’s, I think, probably, the vast majority of our clients have it, and it’s not really a subject that we delve into deep enough in the training for me personally. And I think that’s why I jumped onto this straightaway. (P7, 3‐month, group interview)

While the participant does not elaborate further, they may have been referring to the emotional labour of caring for people who may not have insight into their need for care, or with whom there may be particular challenges in communicating.

#### 3.3.2 Subtheme 2: Reflecting with peers enhances learning

The second subtheme, *Reflecting with peers enhances learning,* explores how the opportunity to share experiences and learn from other homecare workers was highly valued, especially within the context of Covid‐19 and ‘lone‐working’. Participants valued the opportunity for peer learning and support, and some reflected on how this could build on existing support structures within the agency. For one recent starter, the opportunity to speak with peers was new:

This was a bit different, actually meeting other carers (homecare workers) and sharing experiences, that's not something we get otherwise. (P3, 3‐month, individual interview)

In contrast, another homecare worker felt that there were some peer support systems available in the agency, but that these face‐to‐face opportunities were more limited throughout the pandemic:

We usually have a monthly meeting, so we're all there and we discuss all the clients, …but because of Covid, we've not been able to do that, really. (P4, 3‐month, group interview)

One homecare worker hoped that peer support would become more widely available following NIDUS‐professional, and noted that they had raised this with management:

I’ve got a Whatsapp group with some of the other carers but really what we need really is a proper group like yours was, even if it was just once a month… I think the managers, well, they should give us time during our work hours for that kind of thing so, because it could help with our mental health and how we feel about what we do. (P7, 6‐month, individual interview)

The same participant felt that peer support was the main benefit of the implementation groups. Having attended one group catch‐up session and one individual catch‐up session, the participant spoke more positively about the group session:

I think the first one I went to was really useful, it was good to speak with (homecare worker) about how things were going with her and tell you how things were with me. That was the main thing for me. (P7, 6‐month, individual interview)

This aligned with facilitator reflections that individual catch‐ups perhaps felt less valued by participants, and that the group dynamic helped to open up discussions:

They said they had joined the session hoping to hear how the other care workers were getting on but didn’t have much to say one‐to‐one. (Facilitator reflective log – individual catch‐up implementation session)

One homecare worker felt that was there was a less clear purpose for the implementation groups and viewed them primarily as a refresher course, suggesting that the purpose of enabling continued peer support and reflection was less clearly communicated for these sessions:

It was a bit of a refresher really. I’d say some people might find that quite useful, but I didn’t have anything really new to say, really, and we’d done all the training quite recently so it felt quite soon for a refresher session. I’m not sure you needed them really. (P3, 6‐month, individual interview)

These mixed views are reflected in the lower attendance rates we report in the implementation groups.

#### 3.3.3 Subtheme 3: Reflection and perspective taking can improve care

The third subtheme, *Reflection and perspective taking can improve care*, refers to time dedicated to training and the skills taught, enabling participants to reflect on challenging situations from different perspectives and to adapt their response or approach accordingly. All participants spoke positively about the benefits of working through real‐world examples of care. They valued the opportunity to role‐play difficult conversations and discuss examples of challenges they may face in their work with clients and their families:

I thought the challenging role plays that we did were quite good… it’s nice to have some examples and to be prepared for it … so it’s just nice to know how to deal with it, what to do and what’s expected. (P4, 3‐month, group interview)

The DICE model (see Table 1) was consistently remembered by participants, offering a structured model to help them analyse challenging situations. It is specifically developed for use with people living with dementia, who are less able to verbalise their needs and may thus communicate them through their behaviour:

When there’s a challenging situation, because you know care work isn’t straightforward, you can pause, sort of thing, and use the DICE. What was it, now? To describe, to investigate, create the plan and evaluate, sort of thing. So we, sort of, take a pause, and then we think, yes, we need to do that and we need to do this, and then are we asking the right questions. (P4, 3‐month, group interview)

Perspective taking skills were central to the participants’ ability to analyse things differently. They reflected that typically when rushing from one client to the next, there was not often time to reflect:

There’s been more reflecting for me, thinking about what happened for a few minutes after visits since the training, before when the visit was over that was it, on to the next one. Before the training the only reflection was reading notes from the last person, since the training there’s been more conversations with other carers. (P3, 3‐month, individual interview)

In spending more time thinking about the clients’ experience of care, participants drew on their learning from the groups. All shared ideas that they had tried with clients to make care more inclusive and engaging:

Like we say, doing activities with them. I think also getting them involved. Like when we’re making their meals, get them to help as well, so they just don’t sit there and wait for their food, just get them, because it’s sort of active as well. (P1, 3‐month, individual interview)

I gave a lady, I had to wash her hair, and I was giving her a little massage, and I was like, do you like that, and she was like, yes, so she loved it. But it just makes them feel like it’s not just a wash. (P7, 3‐month, group interview)

#### 3.3.4 Subtheme 4: Recognising skills and building confidence

Our fourth subtheme, *Recognising skills and building confidence*, describes the impact of the intervention upon the extent to which participants felt valued, skilled and confident in their role as a homecare worker. All participants discussed how the facilitators had created a welcoming, safe, and relaxed atmosphere. They felt that the facilitators listened, and showed ‘genuine’ interest and care towards homecare workers and clients:

I remember one thing, (facilitator) remembered something that (participant) had said three weeks before, and she would link it back to that, and that was really good to think, gosh, they are actually listening to us as people, and they’re not just there to give a job. (P7, 3‐month, group interview)

There was a sense that some participants had not considered the complexity of their work previously, and appreciated their existing skills being recognised by the facilitators:

I think that was a good breakdown, and actually, what our role involved, because it involved so much more that we weren't aware of, which was nice. (P4, 3‐month, group interview)

Participants all discussed improved confidence in their role as one of the biggest changes that the training had made to them:

I think, without this, I probably would have been a lot more scared, been a lot more, like, no idea what I was doing, to be honest. (P7, 3‐month, group interview)

I think the course gave me more confidence to say “let's do it like this” and to suggest things to other carers. (P3, 3‐month, individual interview)

One participant felt that the intervention and implementation sessions were *‘a good reminder that what you're doing is, you're doing a good job’ (P7, 6‐month interview)*. She reported feeling valued by her clients and their family members but spoke less positively about her day‐to‐day experience of feeling valued within the care agency, and within society more broadly:

The work drains you emotionally and physically… I wish that care work was seen as an actual career, you know, like the NHS, where we’re treated with a bit of respect rather just being seen as dogsbodies. (P7, 6‐month, individual interview)

## 4 DISCUSSION

To the best of our knowledge this is the first manualised person‐centred dementia training intervention, co‐designed for homecare workers who support people living with dementia, to be evaluated in the UK. In this pilot trial, adherence to the intervention and fidelity of delivery were high, indicating that it was acceptable and feasible to deliver, even within the context of the Covid‐19 pandemic.

Our overarching qualitative theme was ‘*Having time and space to reflect is a rare and valued opportunity’*. This explored the different ways in which homecare workers valued the time and space the training afforded to discuss their work and learn new skills. Participants considered the intervention as an opportunity for practical dementia training and peer support that was not available elsewhere, and to reflect on their work in a fast‐paced working environment that left little time for reflection.

Homecare workers found the interactive, group‐based discussions helpful. They shared differing experiences of the level of peer support available within the care agency but agreed that feelings of isolation caused by regular lone‐working had been exacerbated by the pandemic. Although several participants reported a preference for face‐to‐face training, the benefits of peer support were achieved via the group video‐call method.

Our intervention supported participants to develop confidence in their ability to provide thoughtful, person‐centred care and to see challenges commonly arising in homecare from different perspectives. The use of real‐world examples enabled participants to feel more prepared for such circumstances. Facilitator skills were recognised as key to successful intervention delivery.

The intervention is intended to be complementary to the NIDUS‐family intervention (Rapaport et al., 2020) which supports family carers and people living with dementia to live as well as possible at home. Our vision is that the support and care strategies delivered within NIDUS‐family are complemented by accessing homecare, if needed, and that homecare workers are also trained to support the personhood of the person living with dementia. In line with our theoretical model that care should be joined up (with different members of care networks working to common goals) and person‐centred (Lord et al., 2020), we built links between the two interventions; homecare workers were encouraged to bring goals for care that their clients were working on within the NIDUS‐family intervention. Unfortunately, due to the challenges of recruiting in the pandemic, we were not able to test NIDUS‐family alongside NIDUS‐professional as originally planned. We are now doing this in a randomised feasibility trial that is under way.

There is a robust evidence base across different settings (Ballard et al., 2020), and increasingly in homecare settings (Nakanishi et al., 2020; Su et al., 2021), that where staff can be facilitated to build skills in person‐centred care through peer support, case discussion and discussion of care principles, this can improve outcomes for clients (e.g. quality of life, quality of care, reduced agitation). It can also improve retention of care workers, through increased sense of worth, confidence and job satisfaction.

A particular challenge is to develop implementable and sustainable training models, in a setting where staff turnover is high, funding‐limited, time‐pressured (Leverton, 2020; Leverton et al., 2021) and workloads heavy (Su et al., 2021). Participants reflected on how these barriers could be overcome with greater organisational support, for example, through the allocation of protected time for training by employers.

To be scalable, interventions need to be cost‐effective (Rapaport et al., 2020). The shift online necessitated by the pandemic may have led us to one workable solution to this – a remote group intervention.

## 5 LIMITATIONS

This pilot study was conducted within one care agency, with a small sample of homecare workers, and therefore our findings may not be generalisable to other agencies or contexts. Participants were initially approached by the agency training manager who may have approached staff with particular qualities not necessarily representative of the wider workforce, e.g. who were more likely to engage with the training or who were systematically different in some way—whether more highly skilled or in particular need of training—though from facilitator perspectives the group appeared diverse in experience and previous training. The intervention took place mid‐pandemic in the UK and this unusual context may have increased commitment to the group at a time when few other resources were available, or decreased commitment due to multiple, competing demands, personally or professionally.

While four of five participants who attended the main intervention sessions did come to at least one implementation group, attendance was not as high as for the main groups. We will consider how to maintain attendance during the implementation phase in our future trial. Greater clarity around the purpose of this phase (to sustain and utilise peer support at a time when new ways of working, developed through the intervention, are being implemented), clear support from management, and protected time for attendance would be helpful.

We plan to conduct a larger randomised feasibility trial, that should address most of the above limitations.

## 6 CONCLUSION

In this small pilot study, a manualised person‐centred dementia training intervention, specifically co‐designed for homecare workers in the UK, was feasible to deliver and acceptable in the short term, though we identified a challenge in maintaining commitment to the intervention after the formal structured sessions ended. Homecare workers valued the time and space to reflect offered by our intervention. As well as demonstrating the many benefits that such training offers for homecare workers and the nature of the care they provide, our findings highlight the challenging working conditions and the dearth of support for people within this profession, which is critical to not only the wellbeing of people living with dementia and their families, but to society. Our study has implications for policy and practice, in highlighting the value of regular group training and supervision for homecare workers.

## CONFLICT OF INTEREST

The authors declare that there is no conflict of interest.

## AUTHOR CONTRIBUTIONS

All authors made a substantial contribution to this work. DK, KL, LD, PR, ML, JBu, SD and CC all contributed to the study conception, design and material preparation. DK, PR and CC collected the data. DK, KL and CC all coded some of the interview transcripts, organised the data into preliminary themes and led the subsequent analysis. The first draft of the manuscript was written by DK. All authors commented on previous versions of manuscripts and read and approved the final manuscript.

## Supporting information

Supplementary Material

## Data Availability

The qualitative data used and analysed during the current study are available from the corresponding author on reasonable request.

## References

[hsc13737-bib-0001] Ballard, C. , Orrell, M. , Moniz‐Cook, E. , Woods, R. , Whitaker, R. , Corbett, A. , Aarsland, D. , Murray, J. , Lawrence, V. , Testad, I. , Knapp, M. , Romeo, R. , Zala, D. , Stafford, J. , Hoare, Z. , Garrod, L. , Sun, Y. , McLaughlin, E. , Woodward‐Carlton, B. , … Fossey, J. (2020). Improving mental health and reducing antipsychotic use in people with dementia in care homes: The WHELD research programme including two RCTs. Programme Grants for Applied Research, 8(6), 1–98. 10.3310/pgfar08060 32721145

[hsc13737-bib-0002] Braun, V. , & Clarke, V. (2019). Reflecting on reflexive thematic analysis. Qualitative Research in Sport, Exercise and Health, 11(4), 8. 10.1080/2159676X.2019.1628806

[hsc13737-bib-0003] Carter, D. (2016). Fix Dementia Care: Homecare report. alzheimers.org.uk/fixdementiacare.

[hsc13737-bib-0004] Cooper, C. , Cenko, B. , Dow, B. , & Rapaport, P. (2017). A systematic review evaluating the impact of paid home carer training, supervision, and other interventions on the health and well‐being of older home care clients. International Psychogeriatrics, 29(4), 595–604. 10.1017/s1041610216002386 28091355

[hsc13737-bib-0005] Dow, B. , Gaffy, E. , Goh, A. M. Y. , Doyle, C. , Ames, D. , Winbolt, M. , & Scherer, S. (2019). Co‐designing a training programme for paid home carers to deliver dementia specific care to people at home. Alzheimer's & Dementia, 15, 869. 10.1016/j.jalz.2019.06.4611

[hsc13737-bib-0006] Kales, H. C. , Gitlin, L. N. , & Lyketsos, C. G. (2015). Assessment and management of behavioral and psychological symptoms of dementia. BMJ: British Medical Journal, 350, h369. 10.1136/bmj.h369 25731881 PMC4707529

[hsc13737-bib-0007] Lawrence, V. , Fossey, J. , Ballard, C. , Ferreira, N. , & Murray, J. (2016). Helping staff to implement psychosocial interventions in care homes: Augmenting existing practices and meeting needs for support. International Journal of Geriatric Psychiatry, 31(3), 284–293. 10.1002/gps.4322 26192078

[hsc13737-bib-0008] Leverton, M. (2020). “You can’t just put somebody in a situation with no armour”. An ethnographic exploration of the training and support needs of homecare workers caring for people living with dementia. Dementia, 1–24. 10.1177/14713012211023676 PMC8678657 34111969

[hsc13737-bib-0009] Leverton, M. , Burton, A. , Beresford‐Dent, J. , Rapaport, P. , Manthorpe, J. , Azocar, I. , Giebel, C. , Lord, K. , & Cooper, C. (2021). Supporting independence at home for people living with dementia: A qualitative ethnographic study of homecare. Social Psychiatry and Psychiatric Epidemiology, 56(12), 2323–2336. 10.1007/s00127-021-02084-y 33893821 PMC8558284

[hsc13737-bib-0010] Livingston, G. , Barber, J. , Marston, L. , Stringer, A. , Panca, M. , Hunter, R. , Cooper, C. , Laybourne, A. , La Frenais, F. , Reeves, S. , Manela, M. , Lambe, K. , Banerjee, S. , & Rapaport, P. (2019). Clinical and cost‐effectiveness of the Managing Agitation and Raising Quality of Life (MARQUE) intervention for agitation in people with dementia in care homes: A single‐blind, cluster‐randomised controlled trial. Lancet Psychiatry, 6(4), 293–304. 10.1016/S2215-0366(19)30045-8 30872010

[hsc13737-bib-0011] Lord, K. , Beresford‐Dent, J. , Rapaport, P. , Burton, A. , Leverton, M. , Walters, K. , Lang, I. , Downs, M. , Manthorpe, J. , Boex, S. , Jackson, J. , Ogden, M. , & Cooper, C. (2020). Developing the New Interventions for independence in Dementia Study (NIDUS) theoretical model for supporting people to live well with dementia at home for longer: A systematic review of theoretical models and Randomised Controlled Trial evidence. Social Psychiatry and Psychiatric Epidemiology, 55(1), 1–14. 10.1007/s00127-019-01784-w 31679047

[hsc13737-bib-0012] Lord, K. , Kelleher, D. , Ogden, M. , Mason, C. , Rapaport, P. , Burton, A. , Leverton, M. , Downs, M. , Souris, H. , Jackson, J. , Lang, I. , Manthorpe, J. , & Cooper, C. (2021). Co‐designing complex interventions with people living with dementia and their supporters. Dementia, 1–16. 10.1177/14713012211042466 PMC8811333 34969312

[hsc13737-bib-0013] Low, L. F. , Baker, J. R. , Harrison, F. , Jeon, Y. H. , Haertsch, M. , Camp, C. , & Skropeta, M. (2015). The lifestyle engagement activity program (LEAP): Implementing social and recreational activity into case‐managed home care. Journal of the American Medical Directors Association, 16(12), 1069–1076. 10.1016/j.jamda.2015.07.002 26297617

[hsc13737-bib-0014] Moran‐Ellis, J. , Alexander, V. D. , Cronin, A. , Dickinson, M. , Fielding, J. , Sleney, J. , & Thomas, H. (2006). Triangulation and integration: Processes, claims and implications. Qualitative Research, 6(1), 45–59. 10.1177/1468794106058870

[hsc13737-bib-0015] Nakanishi, M. , Endo, K. , Hirooka, K. , Granvik, E. , Minthon, L. , Nägga, K. , & Nishida, A. (2018). Psychosocial behaviour management programme for home‐dwelling people with dementia: A cluster‐randomized controlled trial. International Journal of Geriatric Psychiatry, 33(3), 495–503. 10.1002/gps.4784 28857263

[hsc13737-bib-0016] Nakanishi, M. , Ziylan, C. , Bakker, T. , Granvik, E. , Nagga, K. , & Nishida, A. (2020). Facilitators and barriers associated with the implementation of a Swedish psychosocial dementia care programme in Japan: A secondary analysis of qualitative and quantitative data. Scandinavian Journal of Caring Sciences, 35(2), 430–441. 10.1111/scs.12854 32285513

[hsc13737-bib-0017] Noell, G. H. , Gresham, F. M. , & Gansle, K. A. (2002). Does treatment integrity matter? A preliminary investigation of instructional implementation and mathematics performance. Journal of Behavioral Education, 11(1), 51–67. 10.1023/A:1014385321849

[hsc13737-bib-0018] Polacsek, M. , Goh, A. , Malta, S. , Hallam, B. , Gahan, L. , Cooper, C. , Low, L.‐F. , Livingston, G. , Panayiotou, A. , Loi, S. , Omori, M. , Savvas, S. , Batchelor, F. , Ames, D. , Doyle, C. , Scherer, S. , & Dow, B. (2020). 'I know they are not trained in dementia': Addressing the need for specialist dementia training for home care workers. Health and Social Care in the Community, 28(2), 475–484. 10.1111/hsc.12880 31646701

[hsc13737-bib-0019] Prince, M. , Knapp, M. , Guerchet, M. , Mccrone, P. , Prina, M. , ComasHerrera, A. , Salimkumar, D. (2014). Dementia UK: Update. Alzheimer’s Society (Ed.).

[hsc13737-bib-0020] Rapaport, P. , Burton, A. , Palomo, M. , Griffiths, J. , Kelleher, D. , Leverton, M. , Vickerstaff, V. , Barber, J. , Bird, M. , Budgett, J. , Birch, J. , Rockwood, K. , Downs, M. , Lord, K. , Kales, H. C. , Livingston, G. , Riley, P. , & Cooper, C. (2020). A mixed‐methods feasibility study of a goal‐focused manualised intervention to support people with dementia to stay living independently at home with support from family carers: NIDUS (New Interventions for Independence in Dementia Study) Family. Aging & Mental Health, 25(8), 1463–1474. 10.1080/13607863.2020.1845299 33222498

[hsc13737-bib-0021] Su, H.‐F. , Koo, M. , Lee, W.‐L. , Sung, H.‐C. , Lee, R.‐P. , & Liu, W.‐I. (2021). A dementia care training using mobile e‐learning with mentoring support for home care workers: A controlled study. BMC Geriatrics, 21(1), 126. 10.1186/s12877-021-02075-3 33593287 PMC7885550

[hsc13737-bib-0022] Tong, A. , Sainsbury, P. , & Craig, J. (2007). Consolidated criteria for reporting qualitative research (COREQ): A 32‐item checklist for interviews and focus groups. International Journal for Quality in Health Care, 19(6), 349–357. 10.1093/intqhc/mzm042 17872937

[hsc13737-bib-0023] Yeh, I.‐L. , Samsi, K. , Vandrevala, T. , & Manthorpe, J. (2019). Constituents of effective support for homecare workers providing care to people with dementia at end of life. International Journal of Geriatric Psychiatry, 34(2), 352–359. 10.1002/gps.5027 30430628

