# Supplementary material for: Time to reflect is a rare and valued opportunity; a pilot of the NIDUS‐professional dementia training intervention for homecare workers during the Covid‐19 pandemic
Source: Health Soc Care Community. 2022 Feb 6:10.1111/hsc.13737. Online ahead of print. doi: 10.1111/hsc.13737 (PMC9111618; doi:10.1111/hsc.13737)
Supplement: Supplementary file 1 — Supplementary Material [file HSC-9999-0-s001.docx]

**Appendix 1 – Qualitative Interview/Focus Group Topic Guide**


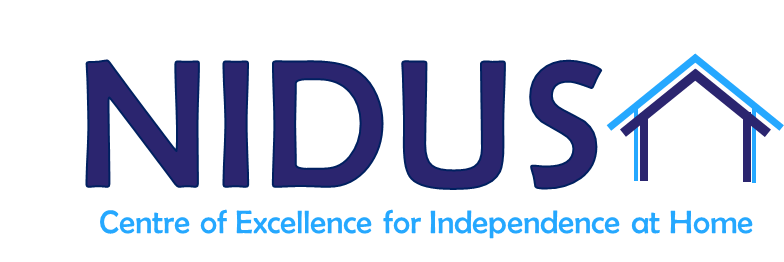


**Supporting independence at home for people with dementia (NIDUS-Professional)**

**Topic Guide**

**Introductions:** Thank you for agreeing to take part in this interview/ focus group. As you know I am a researcher from University College London/University of Bradford and I will be conducting and recording this interview.

**Description of the research:** Two research workers have delivered the NIDUS-professional intervention to you over the past six months. Before we finalise the intervention for testing in a larger research study, we want to ask you for your feedback on your experiences of receiving it. We will then use your suggestions to make further changes to the intervention manual and training for researchers delivering the intervention.

**Q. What did you think about the sessions in general?**

**Prompts:**

- - *What did you like best?*
  - *What did you find most useful?*
  - *What changes were you and your relative able to make as a result of the intervention?*
  - *Was there anything you did not like?*
  - *Was there anything important that you feel was missing?*
  - *Were the sessions easy to understand?*
  - *What would you like to see changed if anything?*

**Q. What did you think about the content of the sessions?**

**Prompts:**

- - *What did you like best about the content?*
  - *Which topics did you find most useful?*
  - *How did the content help you/ your agency change how you delivered care or supported each other?*
  - *Were there any topics that you did not like?*
  - *Were there any important topics that you feel were missing?*
  - *Was the content easy to understand?*
  - *What would you like to see changed about the content if anything?*

**Q. What did you think about the structure of the sessions?**

**Prompts:**

- - *What did you think about the number of sessions offered?*
  - *What did you think about the length of each session?*
  - *What did you think about the length of the overall intervention (6 months)?*
  - *How did the implementation period (when the research team stayed in touch with you after the end of the training sessions) work out?*
  - *What did you think about trying things out/ making changes between sessions?*
  - *Is there anything you would like to change about the structure of the sessions?*

**Q. Do you have any feedback regarding the layout or design of the materials?**

- *How did you find the layout of the materials?*
- *Were the materials easy to read and follow e.g. amount of text on the page, the font, the colours?*
- *How did you find the case studies and examples? Were they helpful?*
- *What did you think of the pictures and images? Would you like to see more, fewer, anything different?*
- *How did the layout/design help you/ your relative to make changes?*
- *How did the layout/design fit with your goals?*

**Q To what extent do you think the intervention could be useful to you in the future, now that the research study has ended?**

**Prompts:**

- *What would help you use it in the future?*
- *What might stop you from using it in the future?*

**Q. Before we finish, is there anything else you would like to mention that we have not already covered?**

**Thank you for your time and for taking part today.**
